# Gut microbiota profile in children affected by atopic dermatitis and evaluation of intestinal persistence of a probiotic mixture

**DOI:** 10.1038/s41598-019-41149-6

**Published:** 2019-03-21

**Authors:** Sofia Reddel, Federica Del Chierico, Andrea Quagliariello, Simona Giancristoforo, Pamela Vernocchi, Alessandra Russo, Alessandro Fiocchi, Paolo Rossi, Lorenza Putignani, May El Hachem

**Affiliations:** 1 Human Microbiome Unit, Bambino Gesù Children’s Hospital IRCCS, Rome, Italy; 2 Dermatology Unit, Bambino Gesù Children’s Hospital IRCCS, Rome, Italy; 3 Unit of Allergology, Bambino Gesù Children’s Hospital IRCCS, Rome, Italy; 4 University Department of Pediatrics, Unit of Immune and Infectious Diseases, IRCCS Bambino Gesù Children’s Hospital, Rome, Italy; 5 Human Microbiome Unit and Parasitology Unit, Bambino Gesù Children’s Hospital IRCCS, Rome, Italy

## Abstract

Atopic dermatitis (AD) has been hypothesised to be associated with gut microbiota (GM) composition. We performed a comparative study of the GM profile of 19 AD children and 18 healthy individuals aimed at identifying bacterial biomarkers associated with the disease. The effect of probiotic intake (*Bifidobacterium breve* plus *Lactobacillus salivarius*) on the modulation of GM and the probiotic persistence in the GM were also evaluated. Faecal samples were analysed by real-time PCR and 16S rRNA targeted metagenomics. Although the probiotics, chosen for this study, did not shape the entire GM profile, we observed the ability of these species to pass through the gastrointestinal tract and to persist (only *B. breve*) in the GM. Moreover, the GM of patients compared to CTRLs showed a dysbiotic status characterised by an increase of *Faecalibacterium, Oscillospira, Bacteroides, Parabacteroides* and *Sutterella* and a reduction of short-chain fatty acid (SCFA)-producing bacteria (i.e., *Bifidobacterium, Blautia, Coprococcus, Eubacterium* and *Propionibacterium*). Taken togheter these results show an alteration in AD microbiota composition with the depletion or absence of some species, opening the way to future probiotic intervention studies.

## Introduction

In the last decades, an increase in allergic diseases has been observed worldwide, especially in westernised countries^1^. Among allergies, atopic dermatitis (AD) is the most common chronic inflammatory skin disease that occurs early in life with a prevalence of 20% in children. The disease has a deep impact on the quality of life of the patient and family^2^. According to the hygiene hypothesis, the effects of modern public health practices, which lower the stimulation of the immune system by microbes, make infants more likely to develop allergic diseases^3,4^. As an extension of the hygiene hypothesis, the “microflora hypothesis of allergic disease” was proposed to underline the role of the gut microbiota (GM) in shaping the development of the host immune system in early life^5^. In fact, the early exposure to gut microbes shifts the Th_1_/Th_2_ balance to a Th_1_ phenotype^6,7^. In contrast, the absence of a normal intestinal bacterial colonisation in atopic diseases, particularly during the mucosal immune system development, pushes the Th_1_/Th_2_ balance towards a Th_2_ response^8,9^. Th_2_ cell-derived mediators, such as IL-4, IL-5 and IL-13, induce immunoglobulin class switching to IgE, thus sustaining the allergy response. Modulation of this response through T-cell deviation to Th_2_ or enhancement of regulatory T-cells (Treg) is a new therapeutic strategy for the prevention and treatment of AD through probiotic administration^10^. Probiotics are defined as living microorganisms that, once ingested, exert health benefits for the host^11^. In the context of atopic diseases, they may act as immune modulators that stimulate Th_1_-mediated responses^12^. The positive effects of probiotics on AD symptoms is already described in literature^13,14^ and, indeed, probiotics administration is routinely employed in clinical practice.

However, recommendations on timing and dose of administration of probiotics in AD have not yet been established. Moreover, not all the studies based on probiotic intake demonstrate the actual ability of probiotics to colonize the GT. This aspect does not allow clinicians to choose the probiotic strain with the certainty that it persists in the GM. In the present study, GM of AD patients and healthy age-matched controls were profiled to identify bacterial biomarkers associated with the disease. The effect of the intake of probiotics composed by *Bifidobacterium breve* and *Lactobacillus salivarius* was evaluated with respect to GM modulation over time to assess the persistence of the probiotic bacteria by quantitative Real-Time PCR (qRT-PCR).

## Materials and Methods

### Study design and sample collection

Nineteen patients in the age range of 0 to 6 years with a diagnosis of AD were prospectively enrolled in the study at the Dermatology Unit of the Bambino Gesù Children’s Hospital in Rome, Italy. Patients took the probiotic (composed of *B. breve* BR03 and *L. salivarius* LS01) twice per day (1 × 10^9^ UFC/dose of each species for 20 consecutive days). Exclusion criteria consisted of treatment with steroids or calcineurin inhibitors, antibiotics intake or gastrointestinal disorders in the four weeks before enrolment and during the follow up. Faecal samples were collected during clinical visits at time T_0_ (no probiotic intake), T_1_ (completion of probiotic intake), T_2_ (60 days after the end of probiotic intake); T_3_ (90 days after the end of probiotic intake).

The treatment was based on antiseptics, emollients, hydration and also focused on therapeutic patient education. In only two patients it was necessary to recurr to topyc steroids during the last two time points.

Seventy-two faecal samples were collected and accompanied by a clinical data diary, SCORAD index values, comorbidities, vaccinations and diet type. The samples were stored at −80 °C at the Human Microbiome Unit of Bambino Gesù Children Hospital in Rome until DNA extraction. Eighteen faecal samples from healthy children of the same age range were also collected as age-matched controls (CTRLs). The criteria for the CTRLs were the absence of chronic diseases or gastrointestinal infections and no antibiotic or probiotic intake in the four weeks before the enrolment. This study was approved by the OPBG Ethics Committee (protocol number 391LB).

All research was performed in accordance with relevant guidelines/regulations. Informed consent was obtained from all parents and/or legal guardians of participants.

### Isolation of *B. breve* BR03 and *L. salivarius* LS01 bacterial strains from the probiotics

The *B. breve* BR03 and *L. salivarius* LS01 bacterial strains were cultured on Columbia agar +5% sheep blood medium (COS, Biomerieux Marcy l’Etoile, France) at 37 °C for 24 h under anaerobic (*B. breve*) or aerobic (*L. salivarius*) conditions. Single colonies were isolated and purified on new COS plates based on their morphology. Bacterial identification was confirmed using a matrix assisted laser desorption/ionization mass spectrometry (MALDI-TOF MS) biotyper and a Microflex LT mass spectrometer (Bruker Daltonics, Bremen, Germany) as described^15^.

### Bacterial DNA extraction from colony and 16S ribosomal locus amplification

DNA was extracted from bacterial colonies using the EZ1 DNA Tissue Kit and automatic extractor biorobot EZ1 according to the manufacturer’s instructions (Qiagen, Hilden, Germany). The entire 16S rDNA locus (1465 bp) was amplified from the extracted DNA using universal primers (27f 5′-AGAGTTTGATCCTGGCTCAG-3′/1492r 5′-ACGGTTACCTTGTTACGACTT-3′). PCR was performed with a reaction mixture containing 5 µl 10X Buffer, 2 µl 2.5 mM MgCl_2_, 2 µl each primer (10 µmol/L), 2 µl dNTPs (10 mmol/L), 1 µl Taq DNA polymerase (5 U/µl) (KAPA Taq PCR kit, KAPA Biosystems, Boston, USA), 5 µl DNA template (10 ng/µl) and molecular-grade H_2_O to a final reaction volume of 50 µl^16^. The amplification protocol consisted of one cycle of initial denaturation at 94 °C for 5 min, 30 cycles of denaturation at 94 °C for 1 min, annealing at 55 °C for 1 min, and extension at 72 °C for 1 min followed by a final extension at 72 °C for 10 min. The resulting amplicons were purified using centrifugal filter units (Amicon Ultra-0.5 mL Centrifugal filters 30 K, Sigma-Aldrich, MO, USA) and quantified using the NanoDrop ND-1000 spectrophotometer (Thermo Fisher Scientific, DE, USA).

### Bacterial DNA cloning and species-specific primer and probe design

The purified PCR products were sequenced and cloned into the PGEM vector following the instructions provided by the pGEM®-T Easy Vector System kit (Promega, Italy) using *Escherichia coli* competent cells as a host. The obtained plasmids, pGEM-BB (pGEM + *B. breve*) and pGEM-LS (pGEM + *L. salivarius*), were extracted (Plasmid Miniprep Kit, Promega, Italy), quantified using the NanoDrop ND-1000 spectrophotometer and diluted. The dilutions, which ranged from 10^6^ to 10^1^ vector copy numbers, were used as standards in the quantitative RT-PCR (qRT-PCR) assays. The cloned fragments of 16S rDNA in the pGEM-BB and pGEM-LS vectors were amplified and sequenced with an automated sequence analyser (Genetic Analyser 3500, Applied Biosystems, CA, USA) using a 50-cm capillary array and a POP-7 polymer (Applied Biosystems) and the BigDye Terminator Cycle Sequencing kit (Applied Biosystems, version 3.1) according to the manufacturer’s instructions. All electropherograms were manually edited for base ambiguity. The obtained FASTA sequences were aligned using CLUSTAL-W software (http://www.ebi.ac.uk/clustalw/) and used for the design of species-specific primers and TaqMan probes (Roche Diagnostics, Mannheim, Germany) (Supplementary Table 1).

### Bacterial DNA extraction from stool samples

Frozen stool samples were thawed at room temperature, and DNA was manually extracted using the QIAmp Fast DNA Stool mini kit (Qiagen, Germany) according to the manufacturer’s instructions. DNA was quantified using the NanoDrop ND-1000. Comparable amounts of DNA (80 ng) from each sample were used in the qRT-PCR assays.

### Targeted-metagenomics

The V1–V3 regions (520 bp) of the 16S ribosomal RNA (rRNA) locus were amplified and pyrosequenced using a 454-Junior Genome Sequencer (Roche 454 Life Sciences, Branford, USA)^17^. The obtained raw reads were analysed using Quantitative Insights into Microbial Ecology software (QIIME) 1.8.0 software^18^ by demultiplexing, quality score checking, low-length excluding and denoising^19^. Sequences were grouped into operational taxonomic units (OTUs) by clustering at a threshold of 97% pairwise identity using UCLUST for sequence clustering^20^ and the representative sequences were submitted to PyNAST for sequence alignment^18^. The Greengenes database (v 13.8) was used for OTU matching.

### qRT-PCR

Quantification of *B. breve* and *L. salivarius* in faecal samples was carried out by qRT-PCR using the Light Cycler 480 platform (Roche Diagnostics, Mannheim, Germany). The assays were performed with a 20 µl PCR amplification mixture containing: 10 µl LightCycler 480 Probe Master mix (Roche Diagnostics), 2 µl primers and probes (optimized concentrations, 0.5 µM and 0.1 µM, respectively) (Supplementary Table 1), 3 µl molecular-grade H_2_O and 5 µl DNA template. Each sample was tested in duplicate to ensure data reproducibility. The RT-PCR temperature profile consisted of an initial denaturation at 95 °C for 10 min, 45 amplification cycles at 95 °C for 10 sec, 60 °C for 30 sec and 72 °C for 1 sec followed by a final cooling step at 40 °C for 30 sec. Absolute quantification was performed using the “second derivative maximum method”^21^.

### Statistical analyses

All data were tested for normal distribution using the Shapiro-Wilk normality test. Statistical analyses were computed using the *phyloseq* R package for alpha and beta diversity^22^. Furthermore, the *adonis* function in the *vegan* R package was used to perform the PERMANOVA test on beta diversity with 999 permutations using the “*strata*” argument within the *adonis* function.

The non-parametric Mann-Whitney U-test and Wilcoxon signed-rank test were used to compare the two independent groups (CTRL versus AD at T_0_) and the data for the time points within the AD group, respectively. The linear discriminant effect size (LEfSe) was computed^23^ with α value equal to 0.05 and a logarithmic LDA score threshold of 2.0. The area under the relative operating characteristic (AUROC) test and discriminant analysis (DA) based on univariate ANOVA, Fisher’s coefficient and leave-one-out classification were applied. Correlations between *B. breve* and *L. salivarius* concentrations were determined by the Spearman test using IBM SPSS statistical software (version 21). Only significant p-values (p < 0.05) corrected using the Holm method were considered^24^.

### Metagenomic data open access repository

All sequences and the associated metadata are available at NCBI: Bioprojects: PRJNA439447, gut metagenomic profile from AD patients; PRJNA268064, gut metagenomic profile from healthy subjects (http://www.ncbi.nlm.nih.gov/bioproject/?term=).

## Results

This study included 19 AD patients and 18 CTRLs, age 0 to 6 years. Patient and CTRL characteristics are summarized in Table 1. In particular 12/19 were vaginal delivered, 13/19 were breast-fed and 15/19 were weaned after 6 months.

**Table 1:** Demographic characteristics of patients and healthy individuals enrolled in this study.

	AD group	CTRL group
N	19	18
Male, N	12	11
Female, N	7	7
Age, years (±sd)	2.2 (1.7)	2 (1.4)
BMI (±sd)	16.3 (1.9)	15.95 (1.4)
Vaginal delivery, N	12	n.a
Caesarean section, N	7	n.a
Breastfeeding, N	13	n.a
Formula feeding, N	6	n.a
weaning time <6 months, N	4	n.a
weaning time ≥6 months, N	15	n.a

### Targeted-metagenomics

Eighty-eight faecal samples were analysed by 16S targeted-metagenomics with two samples excluded due to the poor quality of the metagenomic reads, which consisted of 18 baseline (T_0_) and 52 follow-up (19 at T_1_, 17 at T_2_, 16 at T_3_) samples from the AD patients and 18 from the CTRL subjects. From the total set of samples, 239,153 sequencing reads with a mean value of 2,202 sequences for each sample were obtained.

The microbiota biodiversity of the AD and CTRL groups was determined using alpha and beta diversity analyses. In the comparison between the AD and CTRL groups, we tested the effects of the clinical variables (i.e., delivery, neonatal feeding before weaning, weaning time, age, and BMI) on the AD sample distribution. Beta diversity analysis (Bray-Curtis, unweighted and weighted UniFrac metrics) which included all these variables, showed that “age” and “weaning time” greatly influenced the distribution of samples in the principal coordinate analysis (PCoA). Patients under the age of one year (infants) differed enormously from the rest of the sample cohort and formed a significantly separated cluster (Supplementary Fig. 1, Supplementary Table 2). To avoid *bias* in the comparison between the AD and CTRL groups, we excluded patients under the age of 1 year, thus eliminating the age and weaning time effects (Supplementary Table 3). Hence, final analysis included 15 AD (patients older than 1 year of age) and 18 CTRLs.

**Figure 1:**
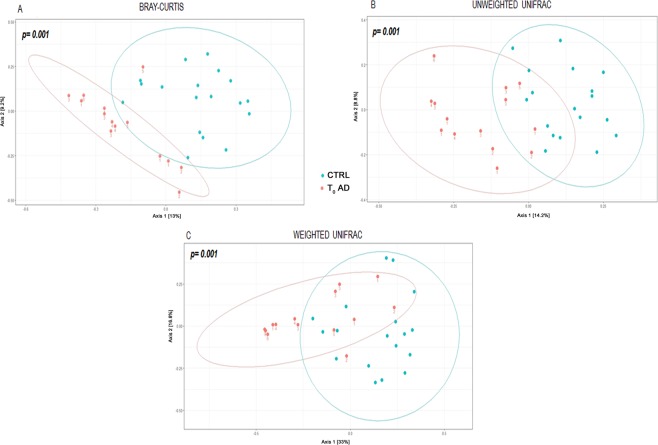
Beta-diversity analysis of AD and CTRL groups. The plots show the first two principal axes for PCoA using Bray-Curtis (**A**), unweighted UniFrac (**B**) and weighted UniFrac (**C**) algorithms. P-values were obtained by PERMANOVA.

To evaluate how OTUs were differentially distributed in the AD and CTRL groups, differences in beta diversity were calculated. A clear separation was observed between the two groups (Fig. 1), which was verified using the PERMANOVA test (p = 0.001 for weighted, unweighted and Bray-Curtis analyses). However, there were no clearly defined clusters for the patients stratified into T_0_ and T_1_–T_3_ groups (PERMANOVA > 0.05) (Supplementary Fig. 2).

**Figure 2:**
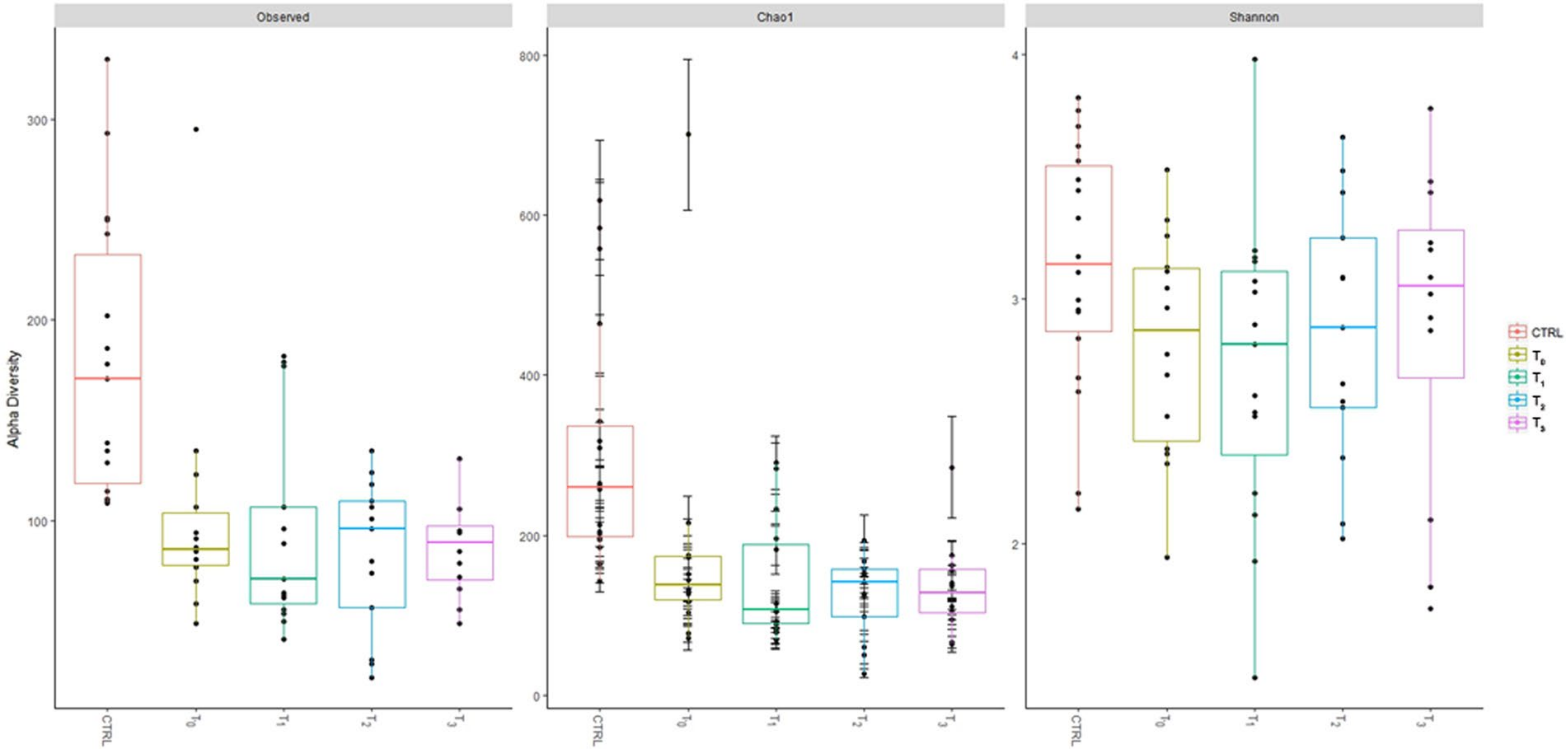
Alpha-diversity measures of Observed, Chao1 and Shannon indexes. Boxes represent the median, 25th and 75th percentiles for AD (time points are indicated) and CTRL groups.

Alpha diversity was calculated with respect to OTU *richness*, *evenness* and *rarity* to understand the ecological differences within the AD and CTRL groups using the Shannon, observed and Chao1 indices, respectively. AD patients showed a significantly lower level of alpha biodiversity according to the observed and ChaoI indices compared to the CTRLs at each time point (T_0_, T_1_, T_2_, T_3_) (Fig. 2, Supplementary Table 3). In addition, the Shannon index revealed less biodiversity in the AD patients, but this result did not reach statistical significance. No statistically significant differences amongst T_0_–T_3_ time point paired comparison were achieved (Supplementary Table 3).

**Figure 3:**
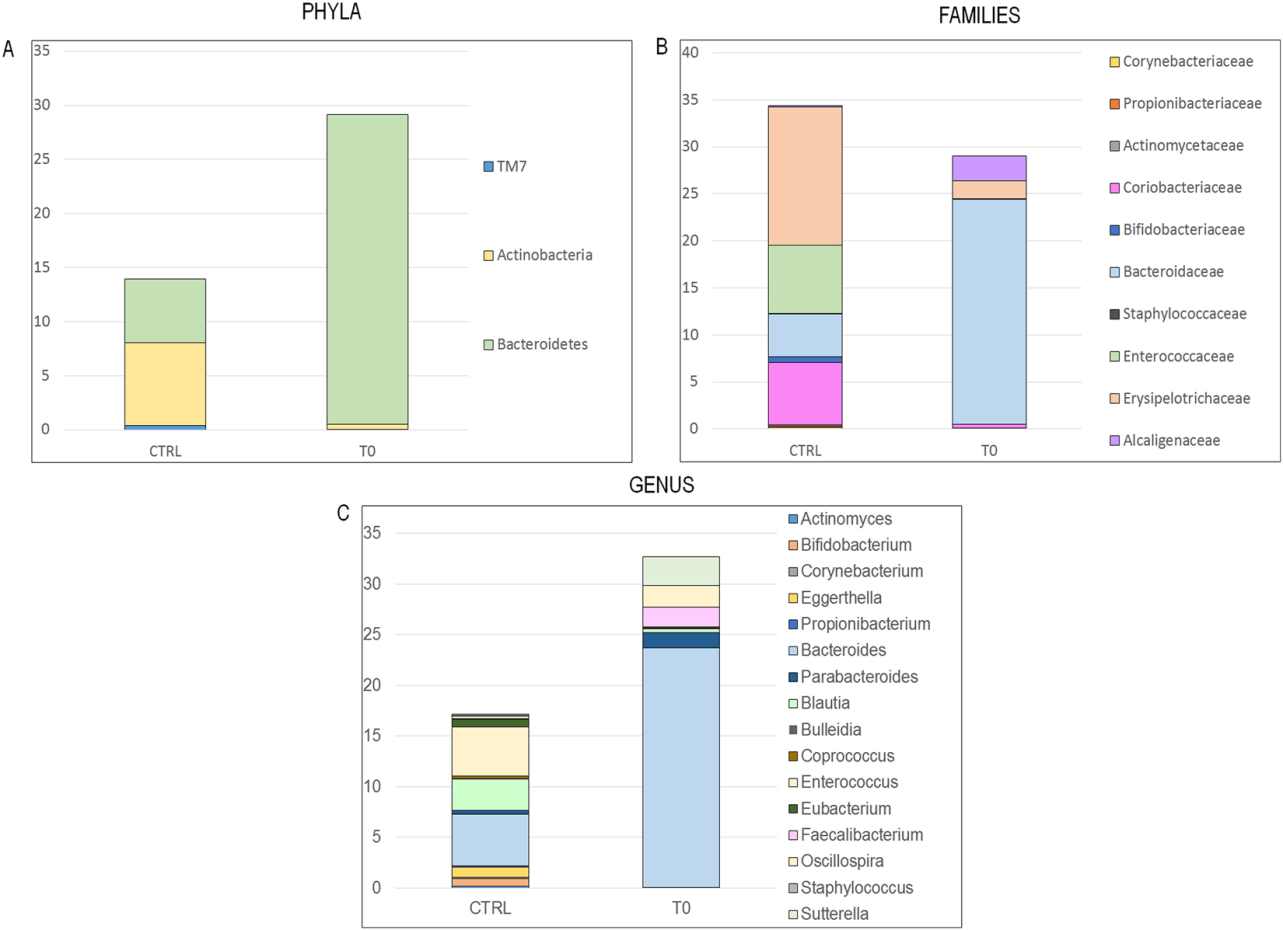
Mann–Whitney-based OTU distribution. The bar graphs represent the average distribution of the OTUs by phylum (**A**), family (**B**) and genus/species (**C**) levels. Only statistically significant OTUs are plotted (p-values were corrected by the Holm method).

To detect differences in OTU composition between AD patients and the CTRLs, we compared time point T_0_ of the AD group (i.e., before the probiotic intake) to the CTRL group. The OTU distribution was investigated at the phyla, family and genus levels. At the phylum level, the Mann-Whitney U-test highlighted the OTU abundance differences, which showed that Bacteroidetes was significantly higher in the T_0_ AD group and Actinobacteria and TM7 were significantly increased in the CTRLs (Fig. 3, Panel A; Supplementary Table 4).

At the family level, the AD group was characterised by lower relative abundances of the Actinobacteria families, such as *Propionibacteriaceae*, *Actinomycetaceae*, *Coriobacteriaceae* and *Bifidobacteriaceae*, and the complete absence of *Corynebacteriaceae* (Fig. 3, Panel B; Supplementary Table 5). Moreover, the Firmicutes families also showed different distributions between the two groups. In particular, *Erysipelotrichaceae* and *Enterococcaceae* were substantially reduced in AD patients, and *Staphylococcaceae* was completely absent. In contrast, these patients were highly enriched in *Bacteroidaceae* (Bacteroidetes) compared to the CTRLs, which accounted for up to 23% of the overall families followed by the Proteobacteria family *Alcaligenaceae* (2.6%) (Fig. 3, Panel B; Supplementary Table 5).

At the genus level, the AD group showed higher relative abundances of *Sutterella (Alcaligenaceae), Bacteroides (Bacteroidaceae), Parabacteroides (Porphyromonadaceae), Oscillospira* and *Faecalibacterium (Ruminococcaceae, Clostridia Class)*, and lower relative abundances of *Eggerthella (Coriobacteriaceae), Propionibacterium (Propionibacteriaceae), Enterococcus (Enterococcaceae), Eubacterium (Erysipelotrichaceae), Actinomyces (Actinomycetaceae), Blautia and Coprococcus (Lachnospiraceae)*. Some OTUs, such as *Staphylococcus (Staphylococcaceae), Bifidobacterium (Bifidobacteriaceae) Corynebacterium (Corynebacteriaceae)* and *Bulleidia (Erysipelotrichaceae)* were completely absent (Fig. 3, Panel C; Supplementary Table 6). To validate our results, we applied LEfSe on our OTU table in the comparison between AD T0 and CTRL. Mann-Whitney U-test and LEfSe analyses showed consistent results (Supplementary Fig. 3).

**Figure 4:**
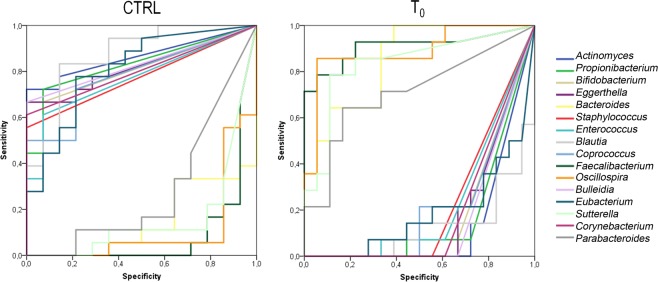
ROC curve plots. The areas under the ROC curves (AUROC) represent the specificity and sensitivity of the 17 selected OTUs to discriminate the T_0_ AD and CTRL groups.

To understand the 16 OTUs belonging group, we applied the average area under the relative operating characteristic (AUROC) test. According to the classification proposed by Swets (Swets 1988), AUROC values ranging from 0.7 to 1 were considered accurate to discriminate between the groups. This analysis showed that the 16 OTUs had discriminatory power. In particular, *Bacteroides*, *Parabacteroides*, *Faecalibacterium*, *Oscillospira* and *Sutterella* were selectively associated with the AD T_0_ group, and *Actinomyces*, *Propionibacterium, Bifidobacterium*, *Eggerthella, Staphylococcus, Enterococcus, Blautia, Coprococcus, Bulleidia, Corynebacterium* and *Eubacterium* were discriminatory for the CTRL group (Fig. 4, Supplementary Table 7).

To estimate the statistical power of these OTUs to act as a classifier for each group, we used the DA model. The DA revealed that 96.9% of the original groups and 84.4% of the cross-validated groups were correctly classified (Supplementary Table 8).

To evaluate the influence of probiotic intake on gut microbiota modulation, we tested the OTU distribution using the pairwise Wilcoxon signed-rank test to compare all time point samples. The test did not find any significant differences at any taxonomic level.

### RT-PCR analysis

**Figure 5:**
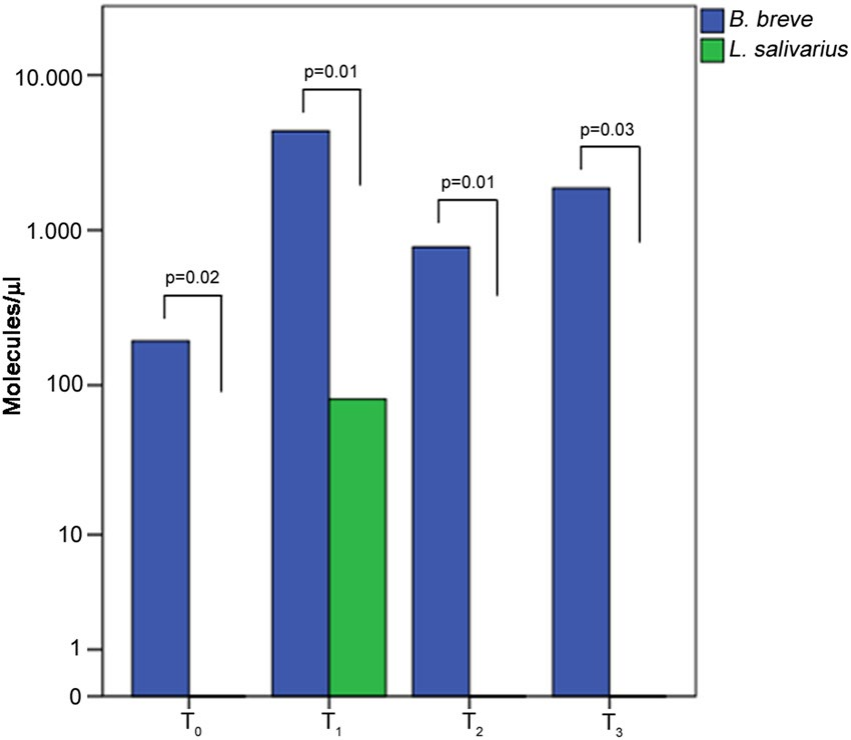
Histograms of *B. breve* and *L. salivarius* levels at different time points. Median values differences between *B. breve* (blue bar) and *L. salivarius* (green bar) levels expressed as molecules/ul at each point of the time-course. P-values were obtained using the Mann–Whitney U test.

RT-PCR was performed on AD patient samples (15 T_0_, 15 T_1_, 14 T_2_ and 11 T_3_ from >1-year-old patients) using primers and probes specific for *B. breve* and *L. salivarius*. The median concentrations (molecules/ul) of the two species were higher at T_1_ than the other time points (Fig. 5). *B. breve* persisted until the latest time point (T_3_) whereas *L. salivarius* decreased to zero by time point T_2_ (Fig. 5). In particular, the *B. breve* concentration differences were statistically significant for the pairwise comparisons between T_0_ and all of the follow-up time points. In contrast, *L. salivarius* concentration achieved significant differences between T_1_ and all the other time points (Supplementary Table 9).

### Correlation analysis

A Spearman’s rank correlation analysis was carried out for each time point to understand the relationship between the *B. breve* and *L. salivarius* levels. Interestingly, at the T_1_ time point, we observed a significant positive correlation between *B. breve* and *L. salivarius*, suggesting that the species cooperated in their tendency to increase (Supplementary Table 10). No other statistically significant correlations were found.

## Discussion

GM dysbiosis has been shown to precede the onset of AD^25^. However, studies on atopic diseases and microbiota are conflicting because both decreased and increased bacterial community diversity have been related to atopy^26^. Our results were consistent with previous findings of low intestinal microbial diversity in AD^27–29^, which supports the theory of ‘microbial deprivation syndromes of affluence^30^. According to this theory, reduced intensity and diversity of microbial stimulation lead to an abnormal immune maturation in early childhood. In fact, limited microbial pressure results in insufficient Th_1_ cell induction and the failure to suppress Th_2_ responses. The switching of the immune stimulation towards a pronounced Th_2_-phenotype is suggested to be a major mechanism to explain allergy development and maintenance^30,31^.

Studies focused on the intestinal microbiota composition in AD children are also contradictory^32–35^.

In our study, we identified some OTUs associated with AD, such as *Faecalibacterium* and *Oscillospira* (Firmicutes), *Bacteroides* and *Parabacteroides* (Bacteroidetes) and *Sutterella* (Proteobacteria).

*Faecalibacterium* genus is generally an indicator of the healthy status of the gut due to its anti-inflammatory effects^36,37^. Low levels of *Faecalibacterium* and in particular of *F. prausnitzii* in the GM have been associated with Crohn’s disease^38^ and AD children^33^. A possible explanation of the high levels of this genus in AD, reported by Song and co-workers^37^, is that the inflamed epithelium with a barrier dysfunction, which is typical in AD, can release nutrients that stimulate the growth of *F. prausnitzii* subspecies that are not short-chain fatty acid (SCFA)-producing. The decreased production of SCFAs, such as butyrate, could lead to further inflammation in the gut epithelium.

*Oscillospira* is a common inhabitant of the GM. It is a butyrate-producing bacterium that utilizes host glycans as growth substrates and contributes to the maintenance of gut health^39^. The role of *Oscillospira* in atopy is not clear. However, our results show that it is strongly associated with AD, which is consistent with the results of Canani and co-workers^32^. Its presence in the GM of AD children could likely be linked to the high abundance of Bacteroidaceae, which produce fermented products that serve as substrates for *Oscillospira* growth^40^.

*Bacteroides* spp. are common inhabitants of the human gut, however their increased presence has been associated with food allergy and other atopic manifestations^41–43^. Indeed, higher levels of *Bacteroides* in atopy could lead to the continuous production of lipopolysaccharides (LPS), the major component of gram-negative cell wall, in the gut, which could trigger an inflammatory response^44^. Moreover, *Bacteroides* species are reported to alter gut permeability^41,45^, a condition observed in AD.

Among the Proteobacteria, *Sutterella* levels were increased in AD children. This genus has been associated with other inflammatory diseases, such as Crohn’s disease and ulcerative colitis, but also with healthy adults^46,47^. Thus, it is still not completely clear if *Sutterella* is involved in inflammation or is a normal inhabitant of the human GM.

Our results revealed that AD GM is characterised by low colonisation of OTUs that have a role in the maintenance of gut health, like *Actinomyces* and *Eggerthella*.

*Actinomyces* spp are members of the normal oral microbiota and have been reported as one of the causative bacteria of dental caries and periodontal diseases^48^. Several studies reported that *Actinomyces* spp. are early colonisers of the healthy infant gastrointestinal tract (GT)^49^.

*Eggerthella* belongs to the Coriobacteria that are common members of the human GM^50^. They are assacharolytic bacteria that produce formate and lactate only from glucose^49^. Until now, only *Eggerthella lenta* and the still not fully characterized *Eggerthella* spp. YY7918 are associated with the human gastrointestinal tract^49^. Morinaga (1988) studied the role of *E. lenta* in the stimulation of the immune system and found that this bacterium is involved in the production of anti-tumour molecules that stimulate natural killer cells^51^. Furthermore, *E. lenta* could have a role in the stimulation of hepatic detoxification and in the inactivation of pharmaceuticals in the gut^52^.

Moreover, AD patients showed a strong reduction of some SCFA-producing bacteria, such as *Bifidobacterium, Blautia*, *Coprococcus*, *Eubacterium* and *Propionibacterium*^53,54^. SCFAs are of particular interest for maintaining host health because they may exert anti-inflammatory effects through several mechanisms, including epithelial integrity (preserving tight junctions) and maintenance of the mucus layer^55^. The resulting low production of SCFAs may be causative of the intestinal barrier dysfunction, increased intestinal permeability and inflammation found in ADs^56^. In particular, *Coprococcus* spp. are butyrate-producing bacteria^49^. Interestingly, Nylund and co-workers reported an inverse correlation between the SCORAD index and the levels of *Coprococcus eutactus*, confirming the role of this particular genus in the amelioration of AD^29^.

*Bifidobacterium* spp. are assumed to be beneficial for human health due to their several effects such as vitamin production, immune system stimulation, inhibition of potentially pathogen bacteria, improvement of food ingredients digestion^57,58^. In the contest of allergic diseases, several studies based on murin and *in vitro* models, have higlighted the potential role of *Bifidobacterium* in reducing inflammation by inducing the production of anti-inflammatory cytokines and suppressing Th_2_ immune response and IgE production^25,59,60^. The absence of *Bifidobacterium* in AD children is consistent with other studies^35,61^ and could lead to a lack of anti-inflammatory effects.

Existing treatments for AD are limited. Therefore, the focus is now to identify alternative strategies. Based on the hygiene hypothesis, probiotics have been proposed as therapeutic and preventive interventions for allergic diseases with the aim to attenuate inflammation^62^. The most used species, belonging to Bifidobacteria and Lactobacilli, have been shown to reduce the risk of AD^63^.

Because of controversial results^64,65^, the influence of probiotics on the prevention or management of AD requires further investigation. In particular, the recommendations on the time of administration and the dose of the probiotic in AD have not been yet drawn up. Huang and collegues^13^ reported a metanalysis of the data available on the topic, highlighting a multitude of administration approaches, including different doses and intake timing. For this reason we investigated the persistence of the two probiotic strains in the GT at defined time points. In our study, we observed an increase of *B. breve* and *L. salivarius* in the stool samples starting from time point T_1_. The significant increase of these species at the end of the probiotic intake, indicated the survival of the bacteria during their transit in the GT. Interestingly, *B. breve* persisted until the last time point (T_3_), while *L. salivarius* did not, suggesting a species-specific survival in the GT.

Furthermore, investigating the action of the probiotic on the AD microbiota profile, we did not observe a significant change in the composition of the GM in AD children after the probiotic intake.

In conclusion, although the probiotics chosen for this study, did not shape the entire GM profile, we can confirm the ability of these species to pass the GT and to persist (only *B. breve*) in the GM. Moreover, our study revealed that the GM of children with AD is characterised by a dysbiotic status with a prevalence of some species such as *Faecalibacterium, Oscillospira, Bacteroides, Parabacteroides and Sutterella*, that can act as possible biomarkers associated to the disease. We also identified a reduction or complete absence of some microbes (*i.e., Bifidobacterium, Blautia*, *Coprococcus*, *Eubacterium* and *Propionibacterium*) with anti-inflammatory effects or involved in immune homeostasis, which might have a protective role against AD. Differences in microbiota composition between AD and CTRL could suggest to take into consideration, in future intervention studies, the species depleted or absent in AD gut microbiota as potential probiotic candidate.

## Supplementary information

Supplementary Material
