# Supplementary material for: Gut microbiota profile in children affected by atopic dermatitis and evaluation of intestinal persistence of a probiotic mixture
Source: Sci Rep. 2019 Mar 21;9:4996. doi: 10.1038/s41598-019-41149-6 (PMC6428866; doi:10.1038/s41598-019-41149-6)
Supplement: Supplementary file 1 — Supplementary Material [file 41598_2019_41149_MOESM1_ESM.docx]

**TITLE**

***Gut microbiota profile in children affected by atopic dermatitis and evaluation of intestinal persistence of a probiotic mixture***

**Authors:** Sofia Reddel^1^, Federica Del Chierico^1^, Andrea Quagliariello^1^, Simona Giancristoforo^2^, Pamela Vernocchi^1^, Alessandra Russo^1^, Alessandro Fiocchi^3^, Paolo Rossi^4^, Lorenza Putignani^1,5,*^ and May El Hachem^2^

**Affiliations:** ^1^Human Microbiome Unit, Bambino Gesù Children’s Hospital IRCCS, Rome, Italy; ^2^Dermatology Unit, Bambino Gesù Children’s Hospital IRCCS, Rome, Italy;

^3^Unit of Allergology, Bambino Gesù Children’s Hospital IRCCS, Rome, Italy;

^4^University Department of Pediatrics, Unit of Immune and Infectious Diseases, IRCCS Bambino Gesù Children's Hospital, Rome, Italy.

^5^Parasitology Unit, Bambino Gesù Children’s Hospital IRCCS, Rome, Italy

* **Corresponding author:** Lorenza Putignani, “Bambino Gesù” Children’s Hospital, Piazza Sant’Onofrio 4, 00165, Rome Italy; phone number: +390668592598; fax number: +3906682904; e-mail address:lorenza.putignani@opbg.net.

**Supplementary Materials**

The supplementary Materials contain: 2 Supplementary Figures (Supplementary Figure 1 and 2) and 10 Supplementary Tables (Supplementary Tables 1-10).

**Supplementary** **Figures**


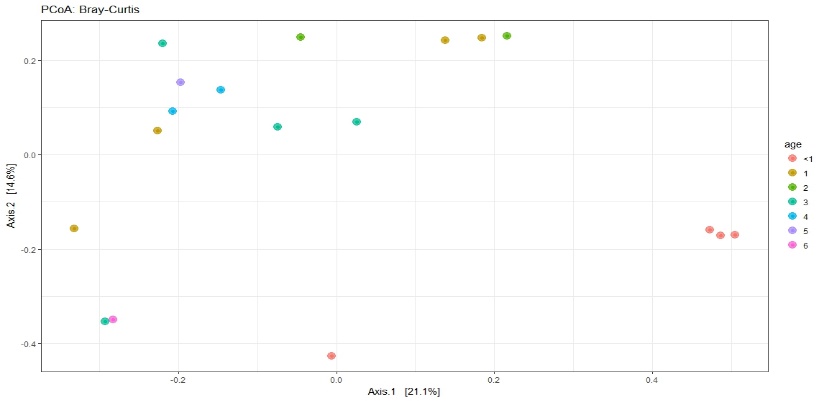

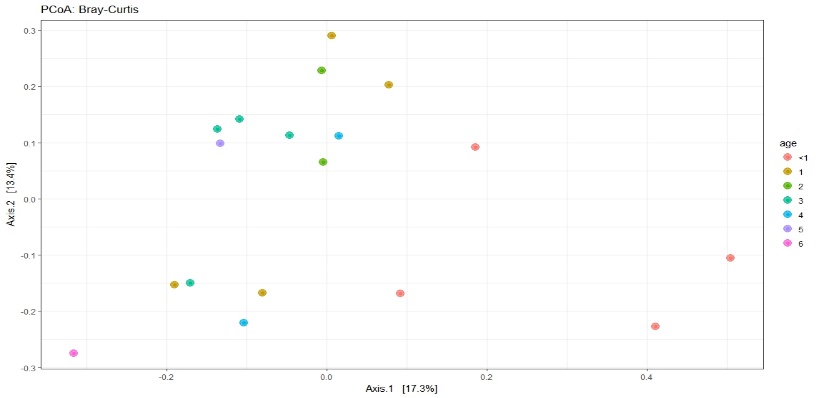


UNWEIGHTED UNIFRAC

BRAY-CURTIS


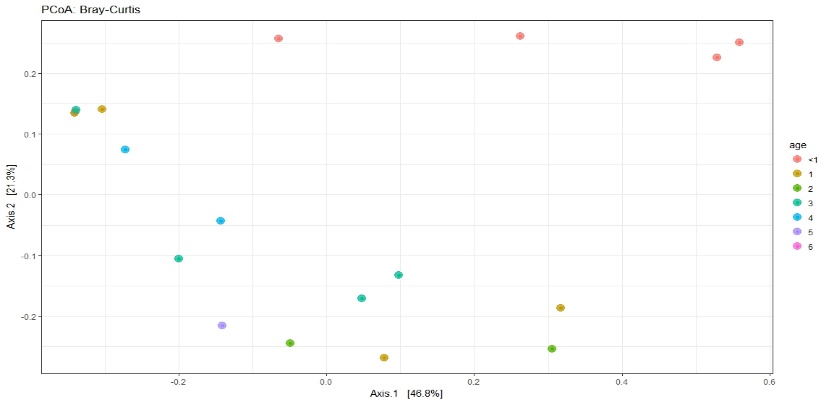


WEIGHTED UNIFRAC

A

B

C

**Supplementary Figure 1. Beta-diversity analysis of AD group at T_0_.** The plots show the first two principal axes for PCoA using Bray-Curtis (A), unweighted UniFrac (B) and weighted UniFrac (C) algorithms. Patients under 1 year of age are indicated with a red circle. P-values obtained by PERMANOVA analyses are reported.


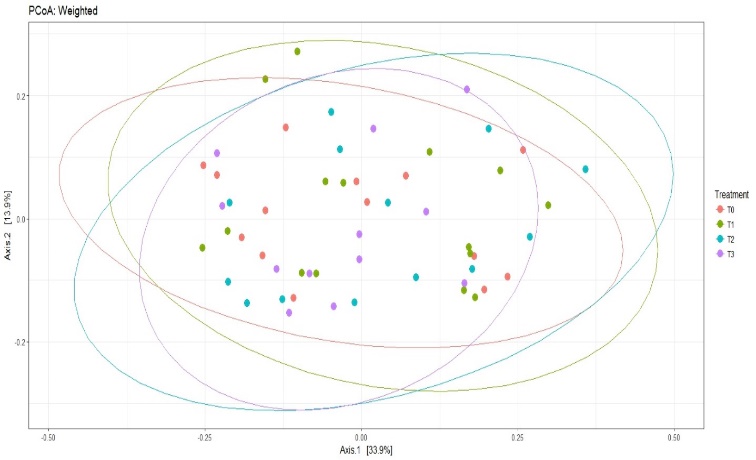

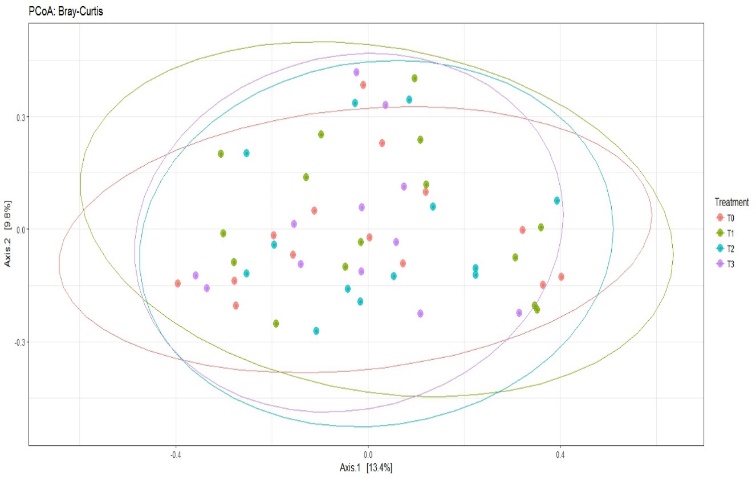

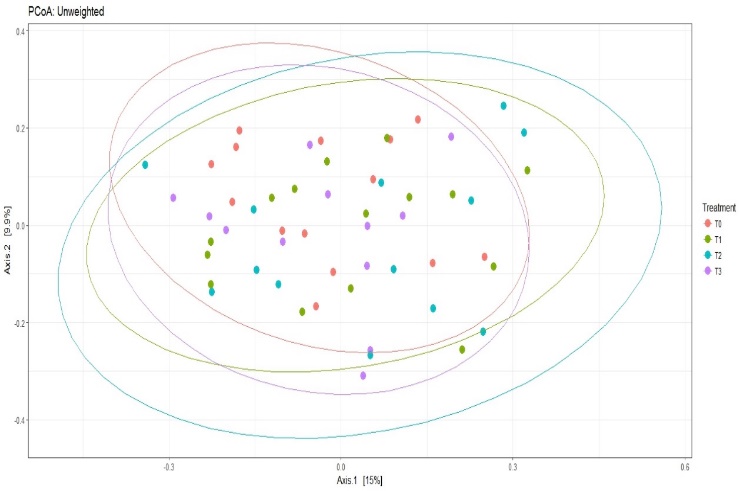


BRAY-CURTIS

UNWEIGHTED UNIFRAC

WEIGHTED UNIFRAC

***p= 0.977***

***p= 0.91***

***p= 0.991***

A

B

C

**Supplementary Figure 2. Beta-diversity analysis applied for all the time points of AD group.** The plots show the first two principal axes for PCoA using Bray-Curtis (A), unweighted UniFrac (B) and weighted UniFrac (C) algorithms. P-values obtained by PERMANOVA analyses are reported.

**Supplementary Figure 3. Linear discriminative analysis (LDA) effect size (LEfSe) analyses of statistically significant Operational Taxonomic Units (OTUs) for AD and** **CTRL T_0_ point.** In green (positive LDA scores) and red (negative LDA scores) are reported OTUs associated with group T0 AD and CTRL, respectively.

**
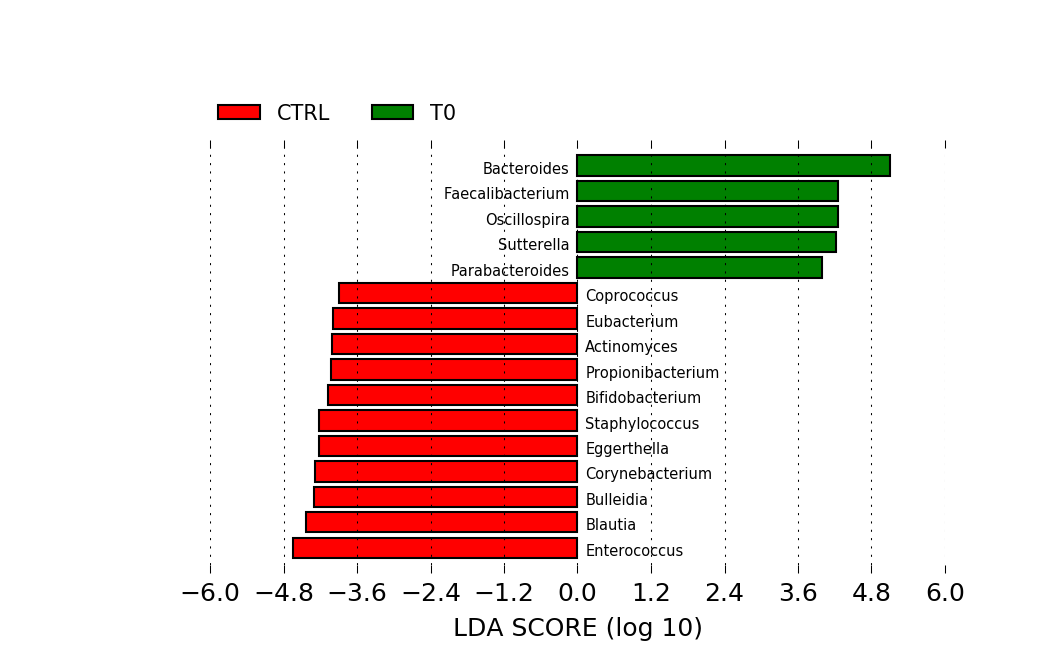
**

**Supplementary Table 1**. Primers and probes designed for *B. breve* and *L. salivarius* for the Real Time assay.

| Species specificity | Primer or probe | Sequence | Product size |
| --- | --- | --- | --- |
| *B. breve* | Fw_Bbreve | 5'-GATGGGGTCGCGTCCTAT-3' | 242 bp |
|  | Rv_Bbreve | 5'-CTCAACACAAAGTGCCTTGCT-3' |  |
|  | P_Bbreve | 5'-FAM CAGCGACGCCGCGTGAGGGATGGAGGCC BHQ-3' |  |
| *L. salivarius* | Fw_Lsalivarius | 5'-AGGTGATGATACGTAGCCGAA-3' | 204 bp |
|  | Rv_Lsalivarius | 5'-GAATGAACAGTTACTCTCACTCGTG-3' |  |
|  | P_Lsalivarius | 5'- HEX CGCCGCGTGAGTGAAGAAGGTCTTCGGATCG BHQ-3' |  |

**Supplementary Table 2.** Permanova test results on beta diversity analyses performed including and excluding infants.

|  | including infants | | | excluding infants | | |
| --- | --- | --- | --- | --- | --- | --- |
|  | **Bray Curtis** | **Weighted UniFrac** | **Unweighted UniFrac** | **Bray Curtis** | **Weighted UniFrac** | **Unweighted UniFrac** |
| Variables | p-value | p-value | p-value | p-value | p-value | p-value |
| Age | **0.002** | **0.016** | **0.044** | 0.220 | 0.098 | 0.311 |
| BMI | 0.35 | 0.35 | 0.889 | 0.297 | 0.315 | 0.900 |
| Neonatal feeding | 0.32 | 0.38 | 0.191 | 0.400 | 0.100 | 0.200 |
| Weaning time | **0.038** | **0.011** | 0.235 | 0.262 | 0.257 | 0.492 |
| Delivery modality | 0.644 | 0.583 | 0.422 | 0.400 | 0.501 | 0.159 |

Values in bold are statistically significant.

**Supplementary Table 3**. Wilcoxon signed rank test with p-values corrected for Holm method.

| **Groups** | **Observed**  ***p value*** | **Chao1**  ***p value*** | **Shannon**  ***p value*** |
| --- | --- | --- | --- |
| T_0_ *vs* CTRL | 0.0027 | 0.0026 | 1.0000 |
| T_1_ *vs* CTRL | 0.0027 | 0.0019 | 0.5984 |
| T_2_ *vs* CTRL | 0.0013 | 0.0000 | 1.0000 |
| T_3_ *vs* CTRL | 0.0008 | 0.0002 | 1.0000 |
| T_4_ *vs* CTRL | 0.0134 | 0.0004 | 1.0000 |
| T_1_ *vs* T_0_ | 1 | 1 | 1 |
| T_2_ *vs* T_0_ | 1 | 1 | 1 |
| T_3_ *vs* T_0_ | 1 | 1 | 1 |
| T_4_ *vs* T_0_ | 1 | 1 | 1 |
| T_2_ *vs* T_1_ | 1 | 1 | 1 |
| T_3_ *vs* T_1_ | 1 | 1 | 1 |
| T_4_ *vs* T_1_ | 1 | 1 | 1 |
| T_3_ *vs* T_2_ | 1 | 1 | 1 |
| T_4_ *vs* T_2_ | 1 | 1 | 1 |
| T_4_ *vs* T_3_ | 1 | 1 | 1 |

**Supplementary Table 4.** U Mann-Whitney test at phylum level of the CTRL and T_0_ AD groups. For each group relative abundances of phyla are reported.

| **Phylum** | **CTRL** | **T_0_** | **p value** |
| --- | --- | --- | --- |
| Actinobacteria | 7.6463 | 0.4904 | **0.0001** |
| Bacteroidetes | 5.8911 | 28.6427 | **0.0019** |
| TM7 | 0.3810 | 0.0121 | **0.0022** |
| Firmicutes | 71.3766 | 56.6330 | 0.1799 |
| Verrucomicrobia | 0.0910 | 0.5835 | 0.7393 |
| Fusobacteria | 0.0310 | 0.1134 | 0.9815 |
| Synergistetes | 0.0000 | 0.0152 | 1 |
| Cyanobacteria | 0.6310 | 0.0988 | 1 |
| Proteobacteria | 13.9476 | 13.4109 | 1 |

Values in bold are statistically significant.

**Supplementary Table 5.** U Mann-Whitney test at family level of the CTRL and T_0_ AD groups. For each group relative abundances of phyla are reported.

| **Phylum** | **Family** | **CTRL** | **T_0_** | **p value** |
| --- | --- | --- | --- | --- |
| Actinobacteria | Coriobacteriaceae | 6.6091 | 0.3906 | **0.0001** |
| Actinobacteria | Actinomycetaceae | 0.1690 | 0.0061 | **0.0009** |
| Actinobacteria | Corynebacteriaceae | 0.1338 | 0.0000 | **0.0048** |
| Actinobacteria | Bifidobacteriaceae | 0.6570 | 0.0019 | **0.0053** |
| Actinobacteria | Propionibacteriaceae | 0.1281 | 0.1091 | **0.0199** |
| Bacteroidetes | Bacteroidaceae | 4.5471 | 23.9424 | **0.0012** |
| Firmicutes | Erysipelotrichaceae | 14.6941 | 1.8436 | **0.0002** |
| Firmicutes | Enterococcaceae | 7.2549 | 0.0740 | **0.0041** |
| Firmicutes | Staphylococcaceae | 0.0598 | 0.0000 | **0.0105** |
| Proteobacteria | Alcaligenaceae | 0.1168 | 2.6322 | **0.0072** |

Values in bold are statistically significant.

**Supplementary Table 6.** U Mann-Whitney test results at genus level of the CTRL and T_0_ AD groups. For each group relative abundances of phyla are reported.

| **Phylum** | **Genus** | **CTRL** | **T0** | **p value** |
| --- | --- | --- | --- | --- |
| Actinobacteria | *Actinomyces* | 0.18873 | 0.00643 | **0.0002032** |
| Actinobacteria | *Bifidobacterium* | 0.74815 | 0.00195 | **0.000585688** |
| Actinobacteria | *Corynebacterium* | 0.15837 | 0 | **0.000603291** |
| Actinobacteria | *Eggerthella* | 0.98343 | 0.03048 | **0.001548945** |
| Actinobacteria | *Propionibacterium* | 0.15146 | 0.00496 | **0.000507574** |
| Bacteroidetes | *Bacteroides* | 5.06 | 23.657 | **0.000406236** |
| Bacteroidetes | *Parabacteroides* | 0.38068 | 1.50348 | **0.033776008** |
| Firmicutes | *Blautia* | 3.06713 | 0.40691 | **0.000377935** |
| Firmicutes | *Bulleidia* | 0.0874 | 0 | **0.000264829** |
| Firmicutes | *Coprococcus* | 0.22915 | 0.00914 | **0.003943051** |
| Firmicutes | *Enterococcus* | 4.83965 | 0.03558 | **0.002974127** |
| Firmicutes | *Eubacterium* | 0.76044 | 0.09386 | **0.002826059** |
| Firmicutes | *Faecalibacterium* | 0.0981 | 1.97224 | **9.08586E-05** |
| Firmicutes | *Oscillospira* | 0.2153 | 2.16519 | **0.000212178** |
| Firmicutes | *Staphylococcus* | 0.06544 | 0 | **0.001316072** |
| Proteobacteria | *Sutterella* | 0.12753 | 2.77603 | **0.000800181** |

Values in bold are statistically significant.

**Supplementary Table 7.** AUROC values for the OTUs selected for the T_0_ DA and CTRL groups.

|  | T0 | CTRL |  |
| --- | --- | --- | --- |
| Variables | Area | |  |
| *Actinomyces* | 0.135 | **0.865** | |
| *Propionibacterium* | 0.169 | **0.831** | |
| *Bifidobacterium* | 0.179 | **0.821** | |
| *Eggerthella* | 0.179 | **0.821** | |
| *Bacteroides* | **0.853** | 0.147 | |
| *Staphylococcus* | 0.222 | **0.778** | |
| *Enterococcus* | 0.228 | **0.772** | |
| *Blautia* | 0.127 | **0.873** | |
| *Coprococcus* | 0.216 | **0.784** | |
| *Faecalibacterium* | **0.909** | 0.091 | |
| *Oscillospira* | **0.889** | 0.111 | |
| *Bulleidia* | 0.167 | **0.833** | |
| *Eubacterium* | 0.188 | **0.812** | |
| *Sutterella* | **0.837** | 0.163 | |
| *Corynebacterium* | 0.194 | **0.806** | |
| *Parabacteroides* | **0.714** | 0.286 | |

Values in bold are statistically significant.

**Supplementary Table 8.** Discriminant analysis based on the 16 selected OTUs at T_0_ for AD and CTRL groups.

|  | | Groups | Predicted Group Membership | | Total |
| --- | --- | --- | --- | --- | --- |
|  |  |  | CTRL | T_0_ |  |
| Original^a^ | % | CTRL | 100 | 0 | 100 |
|  |  | T_0_ | 7.1 | 92.9 | 100 |
| Cross-validated^b^ | % | CTRL | 83.3 | 16.7 | 100 |
|  |  | T_0_ | 14.3 | 85.7 | 100 |

^a^ 96,9% of original grouped cases correctly classified.

^b^ 84,4% of cross-validated grouped cases correctly classified.

**Supplementary Table 9.** Wilcoxon Signed Ranks Test applied for the time-points comparisons of *B. breve* and *L. salivarius* values.

| *B. breve* | | *L. salivarius* | |
| --- | --- | --- | --- |
| Groups | p value | Groups | p value |
| **T0-T1** | **0.001** | **T0-T1** | **0.003** |
| **T0-T2** | **0.001** | **T1-T3** | **0.011** |
| **T0-T3** | **0.003** | **T1-T2** | **0.023** |
| T1-T3 | 0.057 | T0-T2 | 0.109 |
| T2-T3 | 0.248 | T0-T3 | 0.180 |
| T1-T2 | 0.445 | T2-T3 | 0.180 |

Values in bold are statistically significant.

**Supplementary Table 10.** Spearman’s correlation between *B. breve* and *L. salivarius* amounts at T_1_ sample size (N), Spearman rank probability (Rho) and correlation (R) are shown in the table.

|  | *B. breve vs L. salivarius* |
| --- | --- |
| Rho | **0.683** |
| p value | **0.005** |
| N | 15 |

Values in bold are statistically significant.
